# Supplementary material for: Identification of photoperiod-induced specific miRNAs in the adrenal glands of Sunite sheep (Ovis aries)
Source: Front Vet Sci. 2022 Jul 22;9:888207. doi: 10.3389/fvets.2022.888207 (PMC9354845; doi:10.3389/fvets.2022.888207)
Supplement: Supplementary file 1 [file Data_Sheet_1.ZIP › Supplementary file/miRDeep2 file.zip!/miRDeep2 file/Novel_Structure/SPLP42A1/Novel_660.pdf]

Provisional ID	: NC_019481.2_29800
Score total	: 1
Score for star read(s)	: -1.3
Score for read counts	: 0
Score for mfe	: 0.7
Score for randfold	: 1.6
Score for cons. seed	:
Total read count	: 48
Mature read count	: 48
Loop read count	: 0
Star read count	: 0

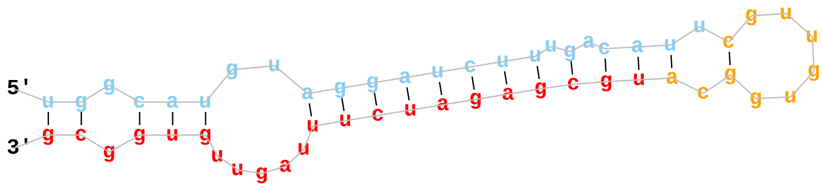

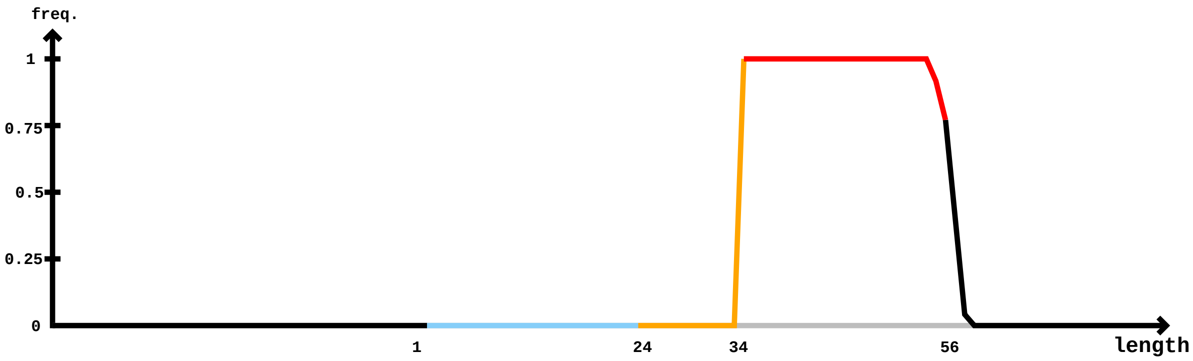

Star Mature

```
5'- gaguuuuuauuuauuuggcuguaccaggucuuaguuguggcauguaggaucuuugacauucguuguggcaugcgagaucuuuaguuguggcguaugaauucuugguugcagcac   -3'    exp
    .................(((((((((((..(((..(((.(((...((((((((.(.(((.(......).))))))))))))....))).)))..)))...))))).))))))..   reads    mm    sample
    ......................................................................ugcgagaucuuuaguugugg........................   1        0     seq
    ......................................................................ugcCagaucuuuaguugugg........................   3        1     seq
    ......................................................................ugcgagaucuuuaguuguggc.......................   4        0     seq
    ......................................................................ugcAagaucuuuaguuguggc.......................   3        1     seq
    ......................................................................ugcgagaucuuuaguuguggcg......................   16       0     seq
    ......................................................................ugcgagaucuuuaguuguggcA......................   5        1     seq
    ......................................................................ugcCagaucuuuaguuguggcgu.....................   1        1     seq
    ......................................................................ugcgagaucuuuaguuguggcgu.....................   12       0     seq
    ......................................................................ugcgagaucuuuaguuguggcAu.....................   1        1     seq
    ......................................................................ugcgagaucuuuaguuguggcgua....................   2        0     seq
```
